# Effect of a high-protein diet with β-cryptoxanthin supplementation on metabolic risk factors, oxidative and inflammatory biomarkers in non-alcoholic fatty liver disease (NAFLD): study protocol for a randomized controlled clinical trial

**DOI:** 10.1186/s13063-018-3014-8

**Published:** 2018-11-16

**Authors:** Fatemeh Haidari, Abdollah Hojhabrimanesh, Bizhan Helli, Seyed Saeid Seyedian, Kambiz Ahmadi-Angali

**Affiliations:** 1 Department of Nutrition, Nutrition and Metabolic Diseases Research Center, Ahvaz Jundishapur University of Medical sciences, Ahvaz, Iran; 2 Gastroenterology Department, Ahvaz Jundishapur University of Medical sciences, Ahvaz, Iran; 3 Faculty of Public Health, Ahvaz Jundishapur University of Medical Sciences, Ahvaz, Iran

**Keywords:** High dose of β-CX supplementation, High protein diet, Biochemical metabolic risk factors, NAFLD

## Abstract

**Background:**

Excessive hepatic fat is associated with increased metabolic risk factors, production of inflammatory factors, and oxidative stress. High protein intake might trigger an increased hepatic lipid oxidation through an increase in hepatic energy expenditure. Furthermore, the majority of randomized controlled trials (RCT) in humans have failed to show whether carotenoids can be used to prevent and treat non-alcoholic fatty liver disease (NAFLD). However, it is notable and contradictory that NAFLD is rapidly escalating in Iran and other countries with lower intakes of fruit and vegetables (as sources of β-cryptoxanthin [β-CX] and carbohydrates) and higher intake of carbohydrates (as an agent of NAFLD); and the effects of β-CX and a high protein diet (HPD) on NAFLD need to be investigated further.

**Methods/design:**

This study will be conducted as a randomized, double-blind, placebo-controlled clinical trial for 12 weeks to receive daily β-CX 6 mg supplementation combined with a HPD on levels of metabolic factors, β-CX, glycemic and lipid profiles, inflammatory factors, adipocytokines, and body composition. Ninety-two eligible patients, aged 18–60 years, of both genders, who are obese and overweight (body mass index [BMI] 25–40 kg/m^2^) will be randomly assigned to four groups as follow: HPD + placebo; normal protein diet + β-CX (NPD + β-CX); HPD + β-CX; and NPD + placebo (control group). Two populations will be analyzed in this work. The intention-to-treat (ITT) population includes all patients who will be randomized, while the per-protocol (PP) population includes all individuals who complete the 12- week intervention (i.e. study completers).

**Discussion:**

Our findings from this trial will contribute to the knowledge of the relationship between β-CX supplementation and a HPD on NAFLD patients and determination of optimal macronutrient ratios without energy restriction.

**Trial registration:**

Iran clinical trials registry, IRCT2017060210181N10. Registered on 20 June 2017.

**Electronic supplementary material:**

The online version of this article (10.1186/s13063-018-3014-8) contains supplementary material, which is available to authorized users.

## Background

Non-alcoholic fatty liver disease (NAFLD) includes a disease spectrum ranging from simple steatosis to non-alcoholic steatohepatitis (NASH), liver fibrosis, cirrhosis, and hepatocellular carcinoma [1]. It is now present in 15–30% of Asians, has a worldwide distribution, and associates with central adiposity, obesity, insulin resistance (IR), metabolic syndrome, cardiovascular diseases (CVD), and type 2 diabetes [2–4]. It occurs in individuals whose alcohol consumption is insignificant (< 10 g per day for women, < 20 g per day for men) and is characterized histologically by at least 5% steatosis and other parenchymal changes, ranging from inflammation to hepatocyte apoptosis/necrosis and to fibrosis [5]. Excessive hepatic fat and visceral adipose tissue mass are also positively associated with systemic inflammation, lipid metabolism disorders [6], serum insulin, free fatty acids, blood glucose, and IR in that each of these causes increased production of inflammatory factors such as TNF-α and oxidative stress [7–9]. C-reactive protein (CRP), TNF-α, and other acute-phase proteins are increased in NAFLD and there is a strong, graded relationship between the histological severity of NAFLD and these markers [10]. The severity of fatty liver is positively correlated with visceral fat accumulation and insulin resistance and may contribute to higher serum liver tests (aspartate transaminase [AST], alanine transaminase [ALT], alkaline phosphatase [ALP], and gamma-glutamyl transferase [GGT]) [11]. There is no precise consent on the effects and features of an optimal nutritional strategy in NAFLD [12]. Common medical interventions include diet therapy to reduce weight, improved nutritional patterns, physical activity, and supplements such as vitamin E and polyunsaturated fatty acids. The goal of each of these interventions is to reduce fat accumulation in the liver or to increase the antioxidant defense [13]. Weight loss may improve NAFLD: although a ≥ 5% weight loss ameliorates steatosis and cardio-metabolic variables, a ≥ 7% weight loss also improves histological disease activity in NASH [14]. Carbohydrate-rich diets increase abdominal fat deposition, free fatty acids, metabolic syndrome, IR, and diabetes [15–19], and associate with obesity [20, 21]. Several mechanisms have been suggested for the potential effects of a high protein diet (HPD; 25–35% of total energy expenditure) versus a normal protein diet (12–18% of total energy expenditure) [22] on NAFLD. Amino acid catabolism is a high energetic process and it is also a well-known fact that taurine increases bile acid conjugation and energy metabolism to prevent diabetes, obesity, and NAFLD [23]. However, well-controlled dietary intervention studies are restricted [24]. β-cryptoxanthin (β-CX) is another component that we want to investigate in this study. β-CX, one of the six major carotenoids routinely measured in human serum (beta-carotene, lycopene, lutein, β-CX, zeaxanthin, and a-carotene), is obtained primarily from citrus fruits [25]. β-CX is also prevalent in corn, peas, and some yellow-colored animal products such as egg yolk and butter. There are many health benefits of a high β-CX diet. Having a high dietary β-CX allows any byproducts to be safely dealt with by more efficient antioxidants in neutralizing free radicals [26]. The recent studies have reported the inverse association of serum GGT, ALT, AST, with serum carotenoids in NAFLD [27–29]. The β-CX is inversely associated with oxidative DNA damage, lipid peroxidation and inflammation, GGT, and insulin resistance [30–35].

There are several reasons for doing this study: heretofore, it is unknown why β-CX as a source of carotenoids can prevent and treat NAFLD. Iranians exhibit lower intake of fruit and vegetables (as source of beta-CX) [36] and higher intake of carbohydrates (as an agent of NAFLD) [37], Observational, in vitro, animal models and human studies suggest that β-CX has greater bioavailability and absorption than alpha- and beta-carotene-rich foods [38]. As the effect of a prolonged intake of high dose of β-CX (6 mg) and a HPD (25% protein of total calorie intake) on NAFLD has received little research attention, a lack of optimal macronutrient ratios without energy restriction, few long-term studies up to now, and few clinical trial studies, we decided to design a clinical RCT to evaluate the effect of a HPD with β-CX supplementation on some metabolic risk factors, oxidative and inflammatory biomarkers, and adipocytokines among NAFLD.

## Methods/design

### Study design

This is a 2 × 2 factorial design that accomplished as a randomized double-blind, placebo-controlled clinical trial. The Standard Protocol Items: Recommendations for Interventional Trials (SPIRIT) 2013 Statement will be followed in this trial (Additional file 1) [36].

### Setting

**Fig. 1:**
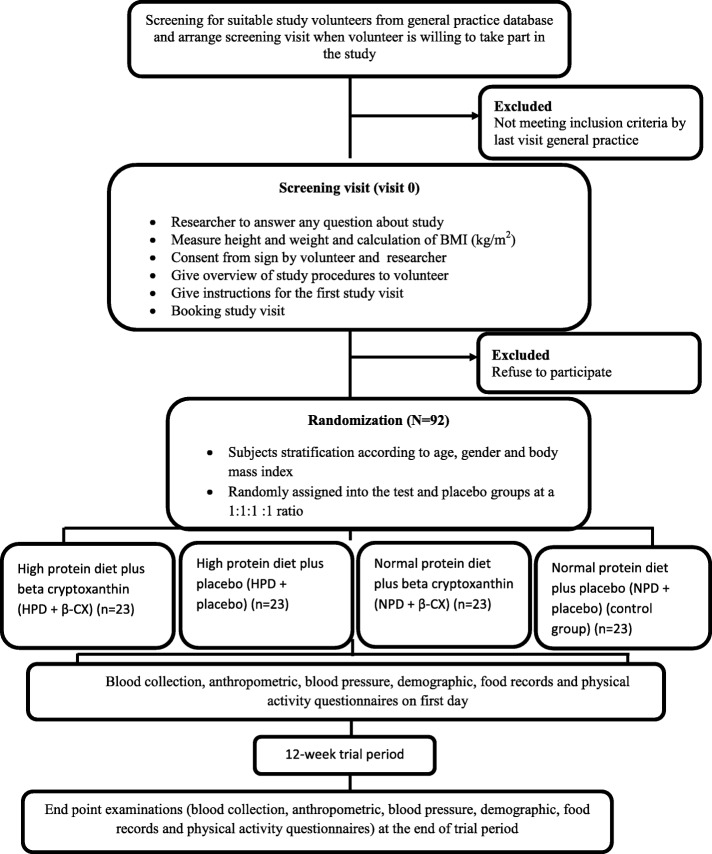
Overview of the study

The proposed clinical trial will be held at the clinic located in the hospital, Ahvaz Jundishapur University of Medical Science, for 12 weeks to assess the efficacy of daily β-CX 6 mg supplementation adjunctive with a HPD in NAFLD individuals. Figure 1 illustrates the overview of the study.

### Participants (inclusion and exclusion criteria)

Patients will be recruited in the study after fulfilling certain criteria, including: overweight and obese (BMI 25–40 kg/m^2^) individuals aged 18–60 years of both genders, existence of NAFLD by ultrasound, NAFLD activity score < 3, and willingness to participate by signing an informed consent document, Patients with viral hepatitis, cirrhosis, Wilson’s disease, pregnancy acute fatty liver, hepatocellular carcinoma, hypothyroidism and a history of chronic liver disease, lipodystrophy, menopause, parenteral nutrition, bladder and bile duct disease, significant weight loss (≥ 10% of body weight in preceding six months) or weight loss due to surgery, congenital metabolic diseases, individuals on antioxidant supplementations, milk thistle, and omega-3 fatty acids in the previous six months, a history of liver-damaging drugs (amiodarone, anti-virus, aspirin, non-steroidal anti-inflammatories, corticosteroids, methotrexate, tamoxifen, tetracycline, valproic acid), alcohol consumption > 20 g/day, calorie intake < 800 kcal or > 4200 kcal a day, pregnancy and lactation, serum ALT levels more than five times the upper limit (maximum limit of 30 for women and 40 for men), or history of cardiovascular and kidney disease (urine analysis albumin ≥ 30 mg/24 h and GFR ≤ 90 mL/min/1.73 m^2^) will be not included.

### Randomization and blinding

Each eligible patient will receive a randomization number which will be determined by a computer-generated schedule. A randomization table will then be generated by the method of random permuted blocks. Persons who will be operationally independent from the study investigator will perform the study randomization. The investigator, clinician prescriber, and patients will be blinded to the treatment condition. To maintain and guarantee blinding, β-CX and placebo will be identical in appearance. Also, all patients will be outpatients and none of the patients will be in contact with each other. Therefore, they cannot compare their diet. Patients’ data collected during this trial will be kept confidential and will be locked in a secure area. Randomization codes of the study will be opened only after all participants complete the study protocol. After the screening phase, participants will be assigned to four equal groups—HPD + placebo; NPD + β-CX; HPD + β-CX; and NPD + placebo (control group)—to receive an outpatient dietary regimen for 12 weeks. The supplements will be bought by Shanghai Tianfu Chemical Ltd. The study will be conducted in accordance with the ethical standards of the responsible Committee on Human Experimentation (institutional and regional) and the guidelines for the design, conduct, and reporting of human intervention studies [37].

### Interventions

**Fig. 2:**
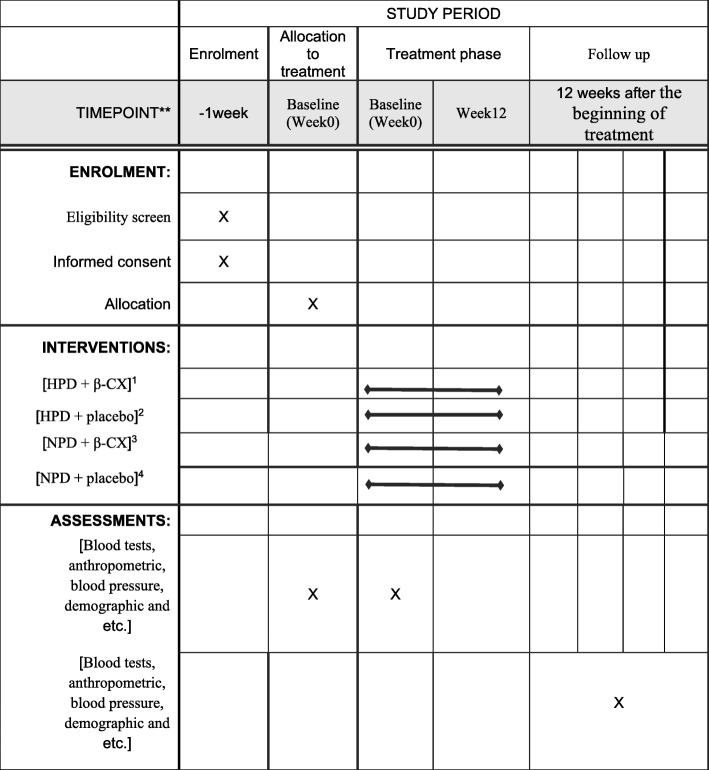
**Schedule for enrollment, intervention, and assessment. ^1^ HPD + β-CX, ^2^ HPD + placebo, ^3^ normal protein diet + β-CX, ^4^ normal protein diet + placebo

According to (SPIRIT) Fig. 2, the study will be conducted for 12 weeks. The visits and the evaluations will be as follows: baseline (visit 0); 0 week; 6 weeks; and 12 weeks. The nutrient goals for the normal protein diet groups are 30% fat, 15% protein, and 55% carbohydrates and for the HPDF are 30% fat, 25% protein, and 45% carbohydrates. The amount of calorie intake will be similar in the four groups and all participants will be instructed to reduce their calorie intake by 500 kcal/day. Throughout this intervention, each patient will be advised not to make any changes in her/his physical activity level (PAL). As the study protocol will require the participants to maintain their pre-study level of physical activity throughout the study period, individuals’ PAL will be assessed at baseline and each follow-up visit using the IPAQ-short form. The supplemented β-CX dose [38, 39] and the recommended protein [17–20, 40] will be set based on previous studies. The placebo will be manufactured to have a similar appearance, shape, weight, taste, and color as the β-CX capsule. The participants will receive either one capsule of β-CX or an identical placebo daily, taken after dinner for a period of 12 weeks.

### Primary outcome measures

Fasting venous blood samples will be taken from patients on the morning of admission and on day 85. Primary outcomes including serum high-sensitive C-reactive protein (CRP) (μg/mL) and adiponectin (μg/mL) will be measured by high-sensitivity enzyme-linked immunosorbent assay (ELISA) with the following characteristics: hs-CRP (BioVendor, Heidelberg, Germany; #RH961CRP01HR); adiponectin (ALPCO Immunoassays, Salem, NH; #47-ADPHU-E01). Plasma insulin (IU/mL) (Pars Azmoon Co., Tehran, Iran), β-CX (HPLC- Column18), and free fatty acids (NEFA C Assay Kit; Wako Chemicals, Neuss, Germany, respectively) as other primary outcomes will be measured by the colorimetric methods. The enzymatic method (Pars Azmoon2 Co., Tehran, Iran) will be used for measuring serum hepatic enzymes (including ALT, AST, and ALP). IR will be assessed by homoeostasis model-insulin resistance index (HOMA-IR) [41], according to the following formulas: HOMA-IR [42] will be calculated using the formula “fasting insulin value fasting blood sugar level/405,” equivalent to the HOMA-formula: HOMA = fasting serum insulin (μU/mL) × fasting plasma glucose (mM/L)/22.5 [42]. HbA1c will be measured by spectrophotometry using the Biorad1 IN2IT devices. Glycemic status (fasting blood glucose) will be determined by the glucose oxidase/peroxidase (GOD-Perid) method using commercially available kits (Pars Azmoon Co., Tehran, Iran).

### Secondary outcome measures

Weight will be measured to the nearest 0.1 kg without shoes while patients are in light clothing. Height will be measured without shoes, with shoulders in a normal position, and will be recorded to the nearest 0.1 cm. BMI will then be calculated as weight in kilograms divided by height in meters squared. Waist circumference will be measured with a non-elastic tape (SECA 203 by SECA GmbH & Co. KG, Hamburg, Germany) at a point midway between the lower border of the rib cage and the iliac crest at the end of normal expiration. Similarly, the hip circumference also will be measured at the widest part of the buttocks at the intertrochanteric level to the nearest 0.1 cm. All anthropometric measures will be taken by trained research assistants using standard equipment according to the standard guidelines [43]. Systolic (SBP) and diastolic blood pressure (DBP) will be measured after at least a 10-min rest with Omron RS6 (also known as HEM-6221-E; Omron Healthcare Co., Ltd., Kyoto, Japan), which is an electronic oscillometric device for BP measurement at the wrist with a cuff size appropriate for wrist circumferences in the range of 13–21 cm. Bioelectrical impendence analysis (BIA) will be performed to assess body composition (BIM4; Impedimed, Brisbane, QLD, Australia). The other anthropometric indices will be calculated using the following equations: waist-to-hip ratio (WHR) = waist (m)/hip (m); waist-to-height ratio (WHtR) = waist (m)/height (m); waist-to-hip-to-height ratio (WHHR) = WHR/height (m); body adiposity index (BAI) = hip (cm)/height (m)^1.5^–18; body shape index (ABSI) = WC (m)/(BMI^2/3^ × height [m]^1/2^) [44]. In addition, lipid parameters (total cholesterol, triglyceride levels, HDL cholesterol, and LDL cholesterol) will be measured. Cholesterol, triglyceride levels, and HDL cholesterol (HDL-C) will be measured with enzymatic colorimetric assays using commercially available kits (Pars Azmoon Co., Tehran, Iran). LDL cholesterol (LDL C) will be estimated based on the Friedewald equation [45]. The malondialdehyde (MDA) will be measured according to a procedure described by the Satoh method [46].

### Dietary assessments

Individuals provided three days of 24-h recall questioner at the beginning and at the end of the study; data were collected by a professional nutritionist and analyzed by Nutritionist IV software (First Databank, San Bruno, CA, USA) modified for Iranian foods.

### Statistical analysis

Two populations will be used in the analyses. The intention-to-treat (ITT) population includes all patients who will be randomized, while the per-protocol (PP) population includes all individuals who complete the 12-week intervention (i.e. study completers). The data will be checked for plausibility by randomly checking the accuracy and completeness and verifying against source data. The variables will be tested for normality using the Kolmogorov–Smirnov test, the Shapiro–Wilk test, and normality plots. All quantitative data will be reported as mean ± standard deviation. Baseline characteristics of participants will be compared among groups using analysis of variance (ANOVA) for parametric data and the Kruskal–Wallis test for non-parametric data. The primary analysis, comparing the effects of treatment on primary outcomes of fatty liver over 12 weeks, will be conducted using a generalized linear mixed-models procedure. Treatments and time will be included as fixed effects and the interactions between interventions and time will be tested. If significant main effects or interaction effects are observed, post-hoc analysis with Bonferroni adjustments will be performed. Potential confounding factors and effect modifiers (e.g. baseline age and gender, etc.) will be investigated within the model. Logistic regression will be used to test the multiplicative interaction. Rothman’s synergy index, which would be equal to unity under additively, and less than unity when suggesting antagonism, will be utilized to examine the postulated interaction effect of a HPD and β-CX on fatty liver. The secondary analysis, comparing the effects of treatment on secondary outcomes of fatty liver over 12 weeks, will be conducted using the same procedure. Potential confounding factors and effect modifiers will be investigated within the model. Differences between participants who complete and withdraw from the trial will be analyzed using an independent t test or the Mann–Whitney test for continuous variables (e.g. age) and chi-square for categorical variables (e.g. gender). Associations between severity of fatty liver and nutritional status at baseline will be assessed using regression analysis. McNemar’s test will be used to analyze the binary variables before and after the intervention. Our statistical analyses will be performed by using SPSS 21 (IBM, Armonk, NY, USA) and the results will be considered significant at *P* < 0.05.

### Sample size and sampling

The sample size was calculated based on the primary outcome measures (12-week change in serum adiponectin, CRP, FFA, insulin, serum β-CX, FBS). Using the standard deviations reported in previous studies [17, 20, 47, 48], a maximum sample size of 18 individuals per group will be selected with mean changes of adiponectin as much as 1.3 and standard division of 1.4 g/day and 30% attrition rate. It calculated by pass 11.

To achieve the target sample size, all patients who will come to the gastrology clinic of Ahvaz hospitals will be included in the study if they will be willing to participate in the study considering the inclusion criteria of the study.

### Safety, adverse effects, and monitoring data

There are no side effects of 6 mg/d of β-CX supplementation [38, 39] and the recommended protein [17–20, 40]. This study will also monitor by a Data Monitoring Committee (DMC). All possible adverse events will also be reported to the Ethics Committee of the Ahvaz Jundishapur University of Medical Sciences. In addition, we will be monitoring compliance, gastrointestinal symptoms, and other adverse effects by phone calls and weekly meetings.

## Discussion

In addition to calorie restriction as the main role of reducing fat in the liver, manipulating of macronutrients and supplementation of micronutrients, especially antioxidants, as a part of lifestyle interventions may prevent the progression of fat accumulation in the liver and prevent NAFLD [49]. There is strong evidence from both experimental and animal studies suggesting that a low protein diet can lead to IR, high blood pressure, and lipid abnormalities, all of which are underlying mechanisms involved in NAFLD [50–52]; while dietary fat consumption may play a causative role in NAFLD [7, 53–56]. A HPD may lead to improvement in fatty liver but it has been poorly assessed and most of these studies are mainly limited to animal models [57]. However, well-controlled dietary intervention is limited [24, 58]. In addition to the HPD, β-CX is another component that we want to study in this work. β-CX is an antioxidant and may protect convert free-radical damage to biomolecules including lipids, proteins, and nucleic acids [26]. The β-CX is inversely associated with oxidative DNA damage, lipid peroxidation, and inflammation, GGT, and IR [30–35]. Of six carotenoids, only β-CX is inversely associated with type 2 diabetes [33].

According to the contradictory effects of β-CX and HPD on NAFLD, more long-term studies and clinical RCTs are needed to clarify the effects of carbohydrate restriction and β-CX supplementation on clinical outcome in patients with NAFLD. The findings will help physicians as a hugely important task to prevent the occurrence of NAFLD in people at risk and healthy individuals and will inform about the health impact of taking β-CX supplements and optimal dietary pattern to lose weight and thus avoid some chronic liver diseases. Finally, this study, as far as we are aware, will be the only study of its type to be conducted in Iran and one of the few studies conducted elsewhere in the world.

### Strengths and weaknesses

The main strength of this study is the double-blind placebo-controlled trial as a highest level of research evidence. Other strengths of the study may be listed as follows:

- There are no side effects of 6 mg/d of β-CX supplementation and we expect a high level of patient compliance in this study;
- Measurement of plasma metabolic parameters will suggest to clinicians the best treatment approach of β-CX supplementation and optimal dietary macronutrients distribution on weight loss, liver biochemistry, and IR in patients with NAFLD;
- We will measure the serum β-CX values by HPLC and its association with NAFLD;
- There are few studies of a HPD on NAFLD.

However, the high cost of a HPD and β-CX supplementation within 12 weeks and the likelihood of increased uric acid and other complications of a HPD may be the main weaknesses of this study.

## Trial status

This trial is in the ongoing phase.

## Additional file

Additional file 1: SPIRIT 2013 Checklist: Recommended items to address in a clinical trial protocol and related documents^*^. (DOC 123 kb)

## Abbreviations

ABSI: Body shape index
ALP: Alkaline phosphatase
ALT: Alanine transaminase
ANCOVA: Analysis of covariance
AST: Aspartate transaminase
BAI: Body adiposity index
BIA: Bioelectrical impendence analysis
BMI: Body mass index
CBC: Cell blood count
CR-HPD: Calorie-restricted high protein diet
DBP: Diastolic blood pressure
FBS: Fasting blood sugar
FFA: Free fatty acids
GGT: Gamma-glutamyl transferase
GPPAQ: General practice physical activity questionnaire
HDL: High-density lipoprotein cholesterol
HOMA-IR: Homoeostasis model-insulin resistance index
HPLC: High-performance liquid chromatography
ITT: Intention-to-treat
LDL: Low-density lipoprotein cholesterol
NAFLD: Non-alcoholic fatty liver disease
NASH: Non-alcoholic steatohepatitis
SBP: Systolic blood pressure
TG: Triglyceride
TNF-α: Tumor necrosis factor alpha
WHHR: Waist-to-hip-to-height ratio
WHR: Waist-to-hip ratio
WHtR: Waist-to-height ratio
β-CX: Beta-cryptoxanthin
